# Proinflammatory Cytokines in Prostate Cancer Development and Progression Promoted by High-Fat Diet

**DOI:** 10.1155/2015/249741

**Published:** 2015-02-04

**Authors:** Hua Xu, Meng-bo Hu, Pei-de Bai, Wen-hui Zhu, Sheng-hua Liu, Jun-yao Hou, Zu-quan Xiong, Qiang Ding, Hao-wen Jiang

**Affiliations:** Department of Urology, Huashan Hospital, Fudan University, Shanghai 200040, China

## Abstract

*Background*. We aimed to examine whether proinflammatory cytokines participated in prostate cancer (PCa) development and progression promoted by high-fat diet (HFD). *Methods*. TRAMP (transgenic adenocarcinoma mouse prostate) mice were randomly divided into two groups: normal diet group and HFD group. Mortality rate and tumor formation rate were examined. TRAMP mice were sacrificed and sampled on the 20th, 24th, and 28th week, respectively. Levels of proinflammatory cytokines, including IL-1*α*, IL-1*β*, IL-6, and TNF-*α*, were tested by FlowCytomix. Prostate tissue of TRAMP mice was used for histology study. *Results*. A total of 13 deaths of TRAMP mice were observed, among which 3 (8.33%) were from the normal diet group and 10 (27.78%) from the HFD group. The mortality rate of TRAMP mice from HFD group was significantly higher than that of normal diet group (*P* = 0.032). Tumor formation rate at 20th week of age of HFD group was significantly higher than that of normal diet group (*P* = 0.045). Proinflammatory cytokines levels, including IL-1*α*, IL-1*β*, IL-6, and TNF-*α*, were significantly higher in HFD TRAMP mice. *Conclusions*. HFD could promote TRAMP mouse PCa development and progression with elevated proinflammatory cytokines levels. Proinflammatory cytokines could contribute to PCa development and progression promoted by HFD.

## 1. Introduction

Prostate cancer (PCa) is the most common malignant tumor and the second leading cause of death among men in United States due to cancer [1]. However, PCa has various incidence and mortality rate in the world. Epidemiological study showed that PCa incidence and mortality rate in United States or Europe were higher than those in Asia, including China [2]. Among all the potential risk factors contributing to the difference, great emphasis was laid on diversities in life style besides genetic background. High caloric intake, especially high-fat diet (HFD), is one of the main characteristics of western life style that might play a crucial role in PCa development and progression.

Several epidemiological studies indicated that PCa might be associated with HFD. Increased fat intake was regarded as a major cause of the elevated incidence and mortality rate of PCa, especially advanced PCa [3–5]. Before transgenic mouse of PCa was widely used, some in vivo studies on human PCa cell transplanted rodent models also suggested that HFD acted as a promoter in PCa development and progression [6–8]. Transgenic adenocarcinoma mouse prostate (TRAMP) animal is one of the most widely used mouse model for PCa, which successfully resembles the human PCa development and progression [9]. We have shown TRAMP mouse as a good in vivo model to study the association between HFD and PCa in our previous research [10, 11]. We concluded that HFD could promote TRAMP mice PCa development and progression. Similar findings were reported by Llaverias et al. [12], Bonorden et al. [13], Park et al. [14], and Chang et al. [15]. Although emerging evidence revealed that HFD was closely related with PCa development and progression, no clear mechanism was uncovered yet.

HFD would contribute to accumulation of adipose tissue, which is thought to be a highly active endocrine tissue secreting numerous factors, including growth factors, cytokines, hormone-like molecules, and many other molecules [16]. Obesity was reported to be linked with a subclinical inflammatory state with higher concentration of proinflammatory mediators such as interleukin (IL)-1*β*, IL-6, and tumor necrosis factor (TNF)-*α* in circulation [17–19]. Several studies demonstrated that these abundant proinflammatory cytokines released from adipose tissue could exert a potential impact on the development and progression of many human cancers, including PCa [20, 21]. Then it leads to our proposed project with a view to examine whether proinflammatory cytokines play an important role in PCa development and progression promoted by HFD.

In this study, we would use our well established HFD-fed TRAMP mouse model to preliminarily examine the inflammatory state in TRAMP PCa. We would measure serum levels of proinflammatory cytokines, namely, IL-1*α*, IL-1*β*, IL-6, and TNF-*α* in TRAMP mice, by using a highly sensitive commercial multiplex suspension array technology kit (FlowCytomix) and flow cytometry, in an attempt to define their potential effect in the association between HFD and TRAMP PCa development and progression.

## 2. Materials and Methods

### 2.1. Animals and Diets

All animal studies were approved by the Institutional Animal Care and Use Committee from the Huashan Hospital, Fudan University. Mice were kept on a 12-hour light/dark cycle with ad libitum access to food and water. TRAMP mice were obtained from Jackson Laboratory (Bar Harbor, Maine, USA). Each TRAMP mouse was kept and raised separately in cage with bedding of cork dust. TRAMP mice were monitored every week when water and food were added. Generation of transgenic mice, isolation of mouse-tiptoe DNA, and PCR-based screening assay were performed as previously reported [22]. Mice were randomly separated into two groups at 20 days of age, which were normal diet group and HFD group. Both groups contained 36 TRAMP mice. As shown in Table 1, normal diet consisted of 16% calories from fats, 64% from carbohydrates, and 20% from proteins. HFD was purchased from Puluteng Bio-technology Limited Company, Shanghai, China, comprising 40% calories from fats, 40% from carbohydrates, and 20% from proteins. TRAMP mice's death would be monitored. All the TRAMP mice, including both euthanized ones on schedule and dead ones, would be sampled. But only those euthanized TRAMP mice would be included in tumor formation rate analysis and serum study.

### 2.2. Tissue Preparation

TRAMP mice from both groups were divided into 3 subgroups, which were planned to be euthanized and sampled on the 20th, 24th, and 28th week, respectively, by asphyxiation of CO_2_ under anesthesia by intraperitoneal injection of pentobarbital (5 mg/100 g). Each subgroup was composed of 12 mice. TRAMP mice were required to fast overnight before sacrifice. Blood was taken from the portal vein by 1 mL syringe. Blood was centrifuged at 13,000 rpm for 10 minutes in a refrigerated centrifuge, and serum was collected to a new eppendorf tube. Serum was kept frozen at −80°C for further study. Prostate was sampled and cut into two parts. One was stored frozen at −80°C for later DNA, RNA, and protein extraction (experiments using DNA, RNA, or protein is not included in this paper). The other was immediately fixed in 10% buffered formalin for hematoxylin and eosin (H&E) staining.

### 2.3. Serum Studies

Concentrations (pg/mL) of IL-1*α*, IL-1*β*, IL-6, and TNF-*α* in serum were measured by using a commercial multiplex suspension array technology kit (Mouse IL-1*α*, IL-1*β*, IL-6, and TNF-*α* FlowCytomix simplex kit, eBioscience) and flow cytometry. 25 *μ*L of serum was tested following manufacturer's instructions. MFI from microspheres was acquired with a BD FACSCanto II and analyzed in FlowCytomix Pro2.2.1 software (eBioscience). Concentration of each analyte was obtained by interpolating fluorescence intensity to a 7-point dilution standard curve supplied by the manufacturer. Any value below the limits of detection was given zero for that cytokine.

### 2.4. Prostate Histology Studies

Prostate tissues were fixed in 10% buffered formalin, processed in an alcohol-xylene series, and embedded in paraffin. Sections were cut at 2 *μ*m and stained with hematoxylin and eosin (H&E). H&E stained sections were subjected to histological analysis and were analyzed by pathologists from Department of Pathology, Huashan Hospital, Fudan University.

### 2.5. Statistical Analysis

The results are expressed as mean ± SD. Two-sample *t*-test was used for comparison of cytokine levels between the two groups. Fisher exact test or *χ* ^2^-test was used for comparison of categorical variables, like mortality rate and tumor formation rate. Statistical analysis was performed by SPSS 19.0. The difference is considered statistically significant when the *P* value is <0.05.

## 3. Results

### 3.1. Mortality Rate and Tumor Formation Rate

During our study, 13 deaths of TRAMP mice were observed. The mortality rate of HDF group was significantly higher than that of normal diet group (Table 2, *P* = 0.032). Each TRAMP mouse was kept and raised separately to avoid being bitten and attacked by other mice. The reason of the higher mortality was assumed to be PCa.

Among the 3 dead TRAMP mice which were fed on normal diet, 1 was from 24th week subgroup and the other 2 were from 28th week subgroup. However, among the 10 dead TRAMP mice which were fed on HFD, 4 were from 24th week subgroup and the other 6 were from 28th week subgroup. All the TRAMP mice were sampled including both scheduled euthanized ones and the 13 dead ones. But only those scheduled TRAMP mice were included in tumor formation rate analysis.

Tumor formation rate was also examined. At 20th week of age, significantly higher percentage of PCa was observed in HFD group than in normal diet group (Table 3, *P* = 0.045). However, tumor formation rates at both 24th and 28th week of age were similar between the two groups as shown in Table 3.

### 3.2. Proinflammatory Cytokine Levels in TRAMP Mice

**Figure 1:**
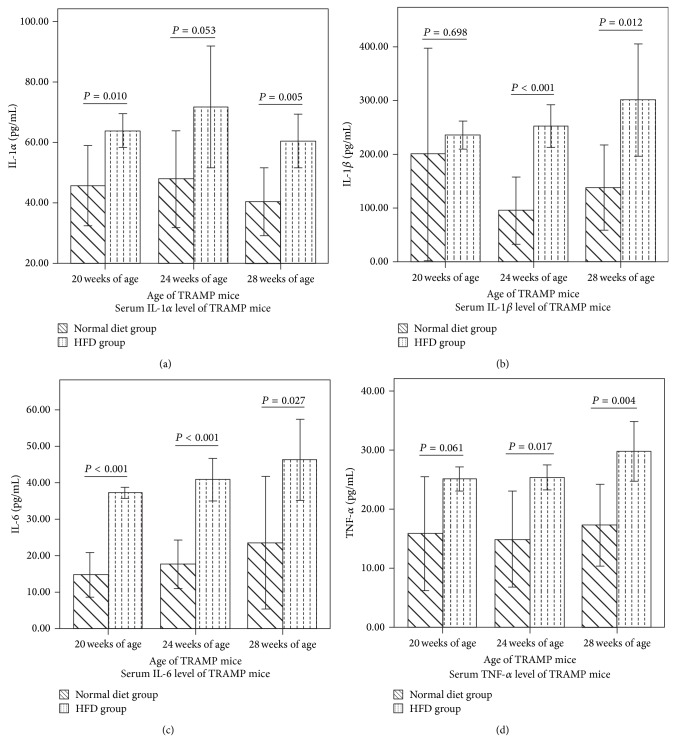
Proinflammatory cytokines levels in TRAMP mice. Two-sample *t*-test was used for comparison of cytokine levels between the two groups. (a) Serum IL-1*α* level in TRAMP mice. (b) Serum IL-1*β* level in TRAMP mice. (c) Serum IL-16 level in TRAMP mice. (d) Serum TNF-*α* level in TRAMP mice.

IL-1*α*, IL-1*β*, IL-6, and TNF-*α* could be detected in TRAMP mice serum as shown in Figure 1.

Figure 1(a) showed IL-1*α* levels in TRAMP mice. A significant increase of IL-1*α* was seen in HFD group of 20th and 28th week of age (*P* = 0.010 and *P* = 0.005, resp.). At 24th week of age, TRAMP mice from HFD group also had moderately higher IL-1*α* than that of TRAMP mice from normal diet group, but the difference was not significant (*P* = 0.053).

Figure 1(b) showed IL-1*β* levels in TRAMP mice. A significant increase of IL-1*β* was seen in HFD group of 24th and 28th week of age (*P* < 0.001 and *P* = 0.012 resp.). Level of IL-1*β* was much higher than IL-1*α* in TRAMP mice serum.

As shown in Figure 1(c), IL-1*β* levels in TRAMP mice from HFD group were significantly higher than those of TRAMP mice from normal diet group at 20th, 24th, and 28th week of age (*P* < 0.001, *P* < 0.001, and *P* = 0.027, resp.). Besides, an increasing trend of IL-6 level was observed as TRAMP mice age increased.

As shown in Figure 1(d), TNF-*α* levels in TRAMP mice from HFD group were significantly higher than those of TRAMP mice from normal diet group at 24th and 28th week of age (*P* = 0.017 and *P* = 0.004, resp.). At 20th week of age, TRAMP mice from HFD group also had moderately higher TNF-*α* than that of TRAMP mice from normal diet group, but the difference was not significant (*P* = 0.061).

## 4. Discussion

Our study indicated that HFD-fed TRAMP mice had increased mortality rate and tumor formation rate, along with increased proinflammatory cytokine levels. Mortality rate of HFD TRAMP mice was significantly higher than that of normal diet ones, indicating that HFD might promote PCa progression and lead to poorer prognosis. Meanwhile, since TRAMP mice were genetically designed to develop PCa eventually, we were just able to discover early elevated tumor formation rate, which implied that HFD could promote PCa development to some extent. Increased serum levels of proinflammatory cytokines, including IL-1*α*, IL-1*β*, IL-6, and TNF-*α*, indicating that chronic inflammation and proinflammatory cytokines might be associated with PCa development and progression promoted by HFD.

A huge change in diet has occurred in many developing countries over the last few decades. This kind of diet is characterized with high portion of refined carbohydrates, saturated fats, and calories and low portion of fruits and vegetables and is often referred to as a “Western diet.” Besides genetic background differences, “Western diet” was listed as one of the main potential risk factors contributing to higher PCa incidence and mortality rate in Western countries than in Asian countries [23]. Among the characteristics of “Western diet,” increased total caloric intake was reported to be positively correlated with PCa risk [24]. Interestingly, fat's potential effect on PCa depended on its type. Walnut oil [25] and fish oil [26] were reported to postpone PCa development or progression. This reminded us that we could not take it for granted that high fat was a risk factor for PCa. Although HFD, accompanied with high caloric intake, could promote PCa development or progression, specific fat or oil type might have different function.

Numerous studies have been carried out, trying to explain the potential relationship between HFD and PCa. One of the hotspots was insulin and insulin-like factor 1 (IGF-1) pathway. HFD was associated with insulin resistance [27], and elevated serum levels of insulin and IGF-1 were supposed to exert promotion of PCa development and progression. Several animal studies showed that PCa xenograft mice fed with HFD had more increased tumor growth and shorter survival time, accompanied with higher serum insulin and IGF-1 levels [28, 29]. Our previous research also demonstrated elevated insulin and IGF-1 levels in HFD-fed TRAMP mouse [10, 11]. These indicated that insulin and IGF-1 pathway might contribute to PCa development and progression promoted by HFD. However, obesity, especially visceral (central) obesity, was associated with proinflammatory state [27]. Though what we examined was not obesity itself, increased caloric consumption is the most important factor for obesity. Proinflammatory states were suggested to be associated with carcinogenesis, including PCa [30]. Cytokines represent key mediators of inflammation and play vital roles in the interaction between inflammation and cancer. IL-1, IL-6, and TNF-*α* are major members of proinflammatory cytokines. According to our study, their levels were elevated to different degrees in HFD-fed TRAMP mice accompanied with promoted PCa development and progression. We would like to extend and talk a little bit about the potential mechanism between these proinflammatory cytokines and PCa.

IL-6 is one of the most popular cytokines studied in PCa. IL-6 combined to its membrane receptor complex, consisting of IL-6 receptor *α* (IL-6R*α*) and glycoprotein 130 (gp130) [31]. Epidemiological studies showed increased serum IL-6 level in patients with castration-resistant PCa [32] and metastatic PCa [33]. Elevated IL-6 level was also associated with PCa biochemical recurrence [34] and poorer overall survival [32]. IL-6 was regarded as an adipokine, since one-third of circulating IL-6 originated from adipose tissue [35]. IL-6 was reported as a paracrine or autocrine growth factor for PCa cells [36]. IL-6 mediated the pathway of Janus kinase (JAK), signal transducer and activator of transcription 3 (STAT3), and mitogen-activated protein kinase (MAPK), which induced androgen receptor-mediated gene expression and eventually induced androgen-independent growth of androgen-dependent PCa cells [37]. IL-6 targeted therapy showed promising prospect in PCa treatment. An anti-IL-6 monoclonal antibody, siltuximab, has been applied in a randomised phase II study (CNTO 328) in metastatic castration-resistant PCa treatment [38].

TNF-*α* is also a pleiotropic cytokine, which is mediated by two distinct receptors named TNF-receptor I (55 kDa, TNFRI) and receptor II (75 kDa, TNFRII) with similar affinity [39]. The expression of TNF-*α* and its receptors has been reported in several human tumors, including PCa [40]. TNF-*α* was shown to be associated with PCa progression [41]. Serum TNF-*α* activity was higher in relapsed PCa patients and these patients had a significantly higher mortality rate than those with undetectable serum TNF-*α* levels [42]. TNF-*α* was also regarded as one of the cytokines which could be secreted by adipose tissue [43]. The biological effect of TNF-*α* was complex and showed contradictory effects. It has been reported that TNF-*α* could exert both proapoptotic and survival/proliferation effects. TNFRI was the major mediator of TNF-*α* activities. TNF-*α*/TNFRI complex was bound to TRADD proteins (TNF receptor associated death domain), which further activated TRAF-2 (TNF receptor associated factor 2) and induced proapoptotic or antiapoptotic signals. The proapoptotic pathway initiated ASK1 (signal regulating kinase), MEK-4 (mitogen activated protein kinase kinase 4), JNK (Jun N-terminal kinase), and latter phosphorylated AP-1 (activator protein-1), which could stimulate apoptosis either directly or by p38 activation [44]. The other antiapoptotic pathway was initiated by NIK (NF-*κ*B-inducing kinase), which could activate NF-*κ*B (nuclear factor *κ*B) and promote survival factors such as bcl-2 and bcl-XL [45].

IL-1 family includes two bioactive ligands (IL-1*α* and IL-1*β*) [46], binding to the same receptors (RI and RII). IL-1*β* is present in the circulation of patients undergoing infectious or inflammatory responses, whereas IL-1*α* is rarely found in the circulation. After IL-1*α* and IL-1*β* was bound to their receptors, the complex could activate IL-1 receptor-associated kinase (IRAK), which is further bound to TNF receptor-associated factor (TRAF)-6. The IRAK/TRAF-6 complex could lead to the activation of NIK [47]. Like TNF-*α*, IL-1 could also get involved in carcinogenesis via NF-*κ*B pathway. The expression of IL-1 family has been reported in several tumors, including PCa, and IL-1 family was reported to be involved in cell proliferation of PCa [48]. Though IL-1*β* level was much higher than IL-1*α*, they were both elevated in HFD TRAMP mice. This again verified that IL-1*β* was more secreted into the microenvironment, while IL-1*α* are more limited and cell-associated.

A great deal of literature has shown that both HFD and inflammation were associated to PCa risk. In our study, we found that HFD might contribute to PCa development and progression via elevated proinflammatory cytokine levels. Inflammation might be one of the mechanisms that explained HFD-fed TRAMP mice had higher mortality and tumor formation rate. Our results further indicated that HFD and inflammation played important roles in PCa development and progression, and diet control as well as anti-inflammation therapy might be helpful to PCa treatment.

Several limitations should be taken into consideration. First, we were only able to assess the mortality and tumor formation rate of TRAMP mice at the moment. We did not examine histological markers of tumor proliferation. While euthanizing TRAMP mice, we sampled prostate as well as other organs, like liver, lung, stomach, pancreas, and testis. We planned to examine whether HFD could influence the metastasis rate of TRAMP mice. Further research and results will be updated and help to draw a better understanding of PCa progression. Second, we plan to extend our study period and study whether HFD could influence TRAMP mice life span and measure proinflammatory cytokines levels dynamically. These survival data together with mortality rate would give a more comprehensive understanding of PCa prognosis. Third, although TRAMP mouse is widely used for PCa research, SV40 T antigen, the foreign oncogene which is inserted and results in the tumorigenesis of prostate, is not normally expressed in human PCa. TRAMP mouse PCa, which is more aggressive, metastatic, and castration resistant, is still a little bit different from human PCa. So the potential mechanism still needs to be further researched, maybe in a better animal model. Besides, we cannot use TRAMP mouse to study the initial phase of PCa carcinogenesis since they are designed to develop PCa eventually.

## Figures and Tables

**Table 1:** Energy and nutrient composition of diets (gm%).

Diet	Normal diet	HFD
Protein	20	22
Fat	7	20
Carbohydrate	64	45
Energy (Kcal/100 g)		
Protein (%)	20	20
Fat (%)	16	40
Carbohydrate (%)	64	40

**Table 2:** Mortality rate of TRAMP mice.

	Normal diet group	HFD group	*P*
	*n* = 36	*n* = 36	
Mortality			
*n*	3	10	0.032
%	8.33	27.78	

**Table 3:** Tumor formation rate of TRMAP mice.

	Normal diet group			HFD group		
	Total number	PCa number	%	Total number	PCa number	%
20th week	12	5	41.67	12	10	83.33^*^
24th week	11	11	100	8	8	100
28th week	10	10	100	6	6	100

^*^ *P* = 0.045; Fisher's exact test.

## References

[1] Siegel R., Ma J., Zou Z., Jemal A. (2014). Cancer statistics, 2014. *CA: A Cancer Journal for Clinicians*.

[2] Rebbeck T. R., Haas G. P. (2014). Temporal trends and racial disparities in global prostate cancer prevalence. *The Canadian Journal of Urology*.

[3] Kristal A. R., Cohen J. H., Qu P., Stanford J. L. (2002). Associations of energy, fat, calcium, and vitamin D with prostate cancer risk. *Cancer Epidemiology Biomarkers and Prevention*.

[4] Fleshner N., Bagnell P. S., Klotz L. (2004). Dietary fat and prostate cancer. *Journal of Urology*.

[5] Lophatananon A., Archer J., Easton D. (2010). Dietary fat and early-onset prostate cancer risk. *British Journal of Nutrition*.

[6] Carroll K. K., Noble R. L. (1987). Dietary fat in relation to hormonal induction of mammary and prostatic carcinoma in Nb rats. *Carcinogenesis*.

[7] Pour P. M., Groot K., Kazakoff K., Anderson K., Schally A. V. (1991). Effects of high-fat diet on the patterns of prostatic cancer induced in rats by *N*-nitrosobis(2-oxopropyl)amine and testosterone. *Cancer Research*.

[8] Tamano S., Rehm S., Waalkes M. P., Ward J. M. (1996). High incidence and histogenesis of seminal vesicle adenocarcinoma and lower incidence of prostate carcinomas in the Lobund-Wistar prostate cancer rat model using N-nitrosomethylurea and testosterone. *Veterinary Pathology*.

[9] Bodmer W. F. (2000). Prostate cancer 2000. *Prostate Cancer and Prostatic Diseases*.

[10] Xu H., Hu M.-B., Bai P.-D., Zhu W.-H., Ding Q., Jiang H.-W. (2014). Will metformin postpone high-fat diet promotion of TRAMP mouse prostate cancer development and progression?. *International Urology and Nephrology*.

[11] Xu H., Jiang W. H., Ding Q. (2014). Insulin-like growth factor 1 related pathways and high-fat diet promotion of transgenic adenocarcinoma mouse prostate (TRAMP) cancer progression. *Actas Urologicas Españolas*.

[12] Llaverias G., Danilo C., Wang Y. (2010). A western-type diet accelerates tumor progression in an autochthonous mouse model of prostate cancer. *The American Journal of Pathology*.

[13] Bonorden M. J. L., Grossmann M. E., Ewing S. A. (2012). Growth and progression of TRAMP prostate tumors in relationship to diet and obesity. *Prostate Cancer*.

[14] Park S.-H., Chang S.-N., Baek M.-W. (2013). Effects of dietary high fat on prostate intraepithelial neoplasia in TRAMP mice. *Laboratory Animal Research*.

[15] Chang S.-N., Han J., Abdelkader T. S. (2014). High animal fat intake enhances prostate cancer progression and reduces glutathione peroxidase 3 expression in early stages of TRAMP mice. *The Prostate*.

[16] Ahima R. S., Flier J. S. (2000). Adipose tissue as an endocrine organ. *Trends in Endocrinology and Metabolism*.

[17] Mantzoros C. S., Moschos S., Avramopoulos I. (1997). Leptin concentrations in relation to body mass index and the tumor necrosis factor-*α* system in humans. *Journal of Clinical Endocrinology and Metabolism*.

[18] Fantuzzi G. (2005). Adipose tissue, adipokines, and inflammation. *Journal of Allergy and Clinical Immunology*.

[19] Wieser V., Moschen A. R., Tilg H. (2013). Inflammation, cytokines and insulin resistance: a clinical perspective. *Archivum Immunologiae et Therapiae Experimentalis*.

[20] Baillargeon J., Platz E. A., Rose D. P. (2006). Obesity, adipokines, and prostate cancer in a prospective population-based study. *Cancer Epidemiology Biomarkers and Prevention*.

[21] Mistry T., Digby J. E., Desai K. M., Randeva H. S. (2007). Obesity and prostate cancer: a role for adipokines. *European Urology*.

[22] Greenberg N. M., DeMayo F., Finegold M. J. (1995). Prostate cancer in a transgenic mouse. *Proceedings of the National Academy of Sciences of the United States of America*.

[23] Lin P.-H., Aronson W., Freedland S. J. (2015). Nutrition, dietary interventions and prostate cancer: the latest evidence. *BMC Medicine*.

[24] Hsieh L. J., Carter H. B., Landis P. K. (2003). Association of energy intake with prostate cancer in a long-term aging study: baltimore longitudinal study of aging (United States). *Urology*.

[25] Kim H., Yokoyama W., Davis P. A. (2014). TRAMP prostate tumor growth is slowed by walnut diets through altered IGF-1 levels, energy pathways, and cholesterol metabolism. *Journal of Medicinal Food*.

[26] Saw C. L. L., Wu T.-Y., Paredes-Gonzalez X., Khor T. O., Pung D., Tony Kong A.-N. (2011). Pharmacodynamics of fish oil: protective effects against prostate cancer in TRAMP mice fed with a high fat western diet. *Asian Pacific Journal of Cancer Prevention*.

[27] Galic S., Oakhill J. S., Steinberg G. R. (2010). Adipose tissue as an endocrine organ. *Molecular and Cellular Endocrinology*.

[28] Freedland S. J., Mavropoulos J., Wang A. (2008). Carbohydrate restriction, prostate cancer growth, and the insulin-like growth factor axis. *Prostate*.

[29] Venkateswaran V., Haddad A. Q., Fleshner N. E. (2007). Association of diet-induced hyperinsulinemia with accelerated growth of prostate cancer (LNCaP) xenografts. *Journal of the National Cancer Institute*.

[30] De Marzo A. M., Platz E. A., Sutcliffe S. (2007). Inflammation in prostate carcinogenesis. *Nature Reviews Cancer*.

[31] Heinrich P. C., Behrmann I., Müller-Newen G., Schaper F., Graeve L. (1998). Interleukin-6-type cytokine signalling through the gp130/Jak/STAT pathway. *Biochemical Journal*.

[32] George D. J., Halabi S., Shepard T. F. (2005). The prognostic significance of plasma interleukin-6 levels in patients with metastatic hormone-refractory prostate cancer: results from cancer and leukemia group B 9480. *Clinical Cancer Research*.

[33] Akimoto S., Okumura A., Fuse H. (1998). Relationship between serum levels of interleukin-6, tumor necrosis factor-*α* and bone turnover markers in prostate cancer patients. *Endocrine Journal*.

[34] Twillie D. A., Eisenberger M. A., Carducci M. A., Hseih W.-S., Kim W. Y., Simons J. W. (1995). Interleukin-6: a candidate mediator of human prostate cancer morbidity. *Urology*.

[35] Fain J. N., Madan A. K., Hiler M. L., Cheema P., Bahouth S. W. (2004). Comparison of the release of adipokines by adipose tissue, adipose tissue matrix, and adipocytes from visceral and subcutaneous abdominal adipose tissues of obese humans. *Endocrinology*.

[36] Azevedo A., Cunha V., Teixeira A. L., Medeiros R. (2011). IL-6/IL-6R as a potential key signaling pathway in prostate cancer development. *World Journal of Clinical Oncology*.

[37] Hobisch A., Eder I. E., Putz T. (1998). Interleukin-6 regulates prostate-specific protein expression in prostate carcinoma cells by activation of the androgen receptor. *Cancer Research*.

[38] Fizazi K., de Bono J. S., Flechon A. (2012). Randomised phase II study of siltuximab (CNTO 328), an anti-IL-6 monoclonal antibody, in combination with mitoxantrone/prednisone versus mitoxantrone/prednisone alone in metastatic castration-resistant prostate cancer. *European Journal of Cancer*.

[39] Loetscher H., Pan Y.-C. E., Lahm H.-W. (1990). Molecular cloning and expression of the human 55 kd tumor necrosis factor receptor. *Cell*.

[40] de Miguel M. P., Royuela M., Bethencourt F. R., Santamaría L., Fraile B., Paniagua R. (2000). Immunoexpression of tumour necrosis factor-*α* and its receptors 1 and 2 correlates with proliferation/apoptosis equilibrium in normal, hyperplasic and carcinomatous human prostate. *Cytokine*.

[41] Michalaki V., Syrigos K., Charles P., Waxman J. (2004). Serum levels of IL-6 and TNF-*α* correlate with clinicopathological features and patient survival in patients with prostate cancer. *British Journal of Cancer*.

[42] Nakashima J., Tachibana M., Ueno M., Miyajima A., Baba S., Murai M. (1998). Association between tumor necrosis factor in serum and cachexia in patients with prostate cancer. *Clinical Cancer Research*.

[43] Bray G. A. (2002). The underlying basis for obesity: relationship to cancer. *Journal of Nutrition*.

[44] Ichijo H., Nishida E., Irie K. (1997). Induction of apoptosis by ASK1, a mammalian MAPKKK that activates SAPK/JNK and p38 signaling pathways. *Science*.

[45] Tamatani M., Che Y. H., Matsuzaki H. (1999). Tumor necrosis factor induces Bcl-2 and Bcl-x expression through NFkappaB activation in primary hippocampal neurons. *The Journal of Biological Chemistry*.

[46] Dinarello C. A. (1994). The interleukin-1 family: 10 years of discovery. *The FASEB Journal*.

[47] Cao Z., Henzel W. J., Gao X. (1996). IRAK: a kinase associated with the interleukin-1 receptor. *Science*.

[48] Ricote M., García-Tuñón I., Bethencourt F. R., Fraile B., Paniagua R., Royuela M. (2004). Interleukin-1 (IL-1*α* and IL-1*β*) and its receptors (IL-1RI, IL-1RII, and IL-1Ra) in prostate carcinoma. *Cancer*.

